# Primary B Lymphocytes Infected with Kaposi's Sarcoma-Associated Herpesvirus Can Be Expanded *In Vitro* and Are Recognized by LANA-Specific CD4^+^ T Cells

**DOI:** 10.1128/JVI.02377-15

**Published:** 2016-03-28

**Authors:** Samantha M. Nicol, Shereen Sabbah, Kevin F. Brulois, Jae U. Jung, Andrew I. Bell, Andrew D. Hislop

**Affiliations:** a Institute of Immunology and Immunotherapy, University of Birmingham, Birmingham, United Kingdom; b Department of Immunobiology, King's College London, London, United Kingdom; c Department of Molecular Microbiology and Immunology, Keck School of Medicine, University of Southern California, Los Angeles, California, USA; d Institute of Cancer and Genomic Sciences, University of Birmingham, Birmingham, United Kingdom

## Abstract

Kaposi's sarcoma-associated herpesvirus (KSHV) has tropism for B lymphocytes, in which it establishes latency, and can also cause lymphoproliferative disorders of these cells manifesting as primary effusion lymphoma (PEL) and multicentric Castleman disease (MCD). T cell immunity is vital for the control of KSHV infection and disease; however, few models of B lymphocyte infection exist to study immune recognition of such cells. Here, we developed a model of B lymphocyte infection with KSHV in which infected tonsillar B lymphocytes were expanded by providing mitogenic stimuli and then challenged with KSHV-specific CD4^+^ T cells. The infected cells expressed viral proteins found in PELs, namely, LANA and viral IRF3 (vIRF3), albeit at lower levels, with similar patterns of gene expression for the major latency, viral interleukin 6 (vIL-6), and vIRF3 transcripts. Despite low-level expression of open reading frame 50 (ORF50), transcripts for the immune evasion genes K3 and K5 were detected, with some downregulation of cell surface-expressed CD86 and ICAM. The vast majority of infected lymphocytes expressed IgM heavy chains with Igλ light chains, recapitulating the features seen in infected cells in MCD. We assessed the ability of the infected lymphocytes to be targeted by a panel of major histocompatibility complex (MHC) class II-matched CD4^+^ T cells and found that LANA-specific T cells restricted to different epitopes recognized these infected cells. Given that at least some KSHV latent antigens are thought to be poor targets for CD8^+^ T cells, we suggest that CD4^+^ T cells are potentially important effectors for the *in vivo* control of KSHV-infected B lymphocytes.

**IMPORTANCE** KSHV establishes a latent reservoir within B lymphocytes, but few models exist to study KSHV-infected B cells other than the transformed PEL cell lines, which have likely accrued mutations during the transformation process. We developed a model of KSHV-infected primary B lymphocytes that recapitulates features seen in PEL and MCD by gene expression and cell phenotype analysis, allowing the study of T cell recognition of these cells. Challenge of KSHV-infected B cells with CD4^+^ T cells specific for LANA, a protein expressed in all KSHV-infected cells and malignancies *in vivo*, showed that these effectors could efficiently recognize such targets. Given that the virus expresses immune evasion genes or uses proteins with intrinsic properties, such as LANA, that minimize epitope recognition by CD8^+^ T cells, CD4^+^ T cell immunity to KSHV may be important for maintaining the virus-host balance.

## INTRODUCTION

Kaposi's sarcoma-associated herpesvirus (KSHV) is one of the two human gammaherpesviruses with oncogenic potential. The virus has tropism for endothelial cells, where it is associated with the development of Kaposi's sarcoma (KS), as well as being tropic for B lymphocytes, where it can cause primary effusion lymphoma (PEL) and multicentric Castleman disease (MCD) (1). The immune response is important for control of KSHV infection, as infected patients whose cellular immune responses are suppressed either for transplantation or due to untreated HIV infection are at an increased risk of developing KS. Importantly, in these scenarios, if immune competence is restored through relaxation of immunosuppression or administration of highly active antiretroviral therapy, respectively, regression of KS lesions can be seen, implying an important role for the T cell response in control of the virus and malignancies (2, 3).

KSHV is known to infect CD19^+^ B lymphocytes (4), but little is known about how KSHV-specific T cell control is exercised over infected B lymphocytes. The only model that has been used to examine T cell recognition of infected B lymphocytes comes from studies using PEL-derived lines as targets. Here, CD8^+^ T lymphocytes were unable to recognize reporter antigens expressed in PELs, which was thought to be related to their low level of expression of the transporter associated with antigen-processing 1 mRNA, disrupting antigen presentation to these effectors (5). CD4^+^ T cells specific for the genome maintenance protein LANA, a protein expressed in all infected cells and malignancies, in most cases showed poor if any recognition of PELs (6). This was a consequence of expression of the KSHV viral IRF3 (vIRF3) gene, which, among other functions, inhibits expression of the major histocompatibility complex (MHC) class II transcriptional transactivator (CIITA), a protein required for the expression of class II and other genes in this antigen-processing pathway (7). Although these studies are performed on cell lines derived from patients with disease, the cell lines have likely accrued mutations and may not resemble B cells in which the virus maintains latency. The ability of KSHV-specific T lymphocytes to respond to KSHV-infected nontransformed B lymphocytes, which are likely to have intact antigen-processing pathways, is so far largely untested.

Several models of primary B cell infection have been developed to study KSHV infection in these cells. The initial studies used CD40 ligand (CD40L) stimulation of B cells to make them receptive to infection (8), while others have cocultured B lymphocytes with virus producer cells to allow direct cell-to-cell virus transfer or have exposed B lymphocytes to concentrated preparations of KSHV (9–12). Such *in vitro*-infected cells express latent transcripts and transiently express selected lytic transcripts and genes, inducing some proliferation of the infected cells, but unlike the related gammaherpesvirus Epstein-Barr virus (EBV), this does not lead to transformation (10). These models have been informative for studying which cytokines may be induced by infection (13) and for examining the potential identity of B lymphocyte targets of KSHV, which are likely IgM heavy chain, Igλ light chain, CD27^+^ cells (10).

However, the analysis of immune recognition of *ex vivo*-infected B cells has been limited, with studies suggesting that CD4^+^ T cells may suppress spontaneous lytic replication and encourage latency (11). This mechanism required the infected cell to be in contact with activated CD4^+^ T cells, but was independent of MHC restriction, and so how such restriction might operate *in vivo* is not clear. Furthermore, how *ex vivo*-infected cells that have stably entered latency may be controlled by KSHV-specific immune effectors has not been tested. To examine these questions, we developed a KSHV B cell infection model in which we expanded infected cells to ask what viral genes are expressed in infected cells, how infection may modulate immune receptor expression, and whether antigen-specific T cells can recognize these targets.

## MATERIALS AND METHODS

### Tonsil cell preparations and infections.

Tonsil specimens were obtained from patients undergoing routine tonsillectomy to treat chronic tonsillitis. The patients were adolescents or young adults, and their tonsils were not inflamed at the time of surgery. All participants gave written informed consent in accordance with the Declaration of Helsinki; ethical approval was granted by the South Birmingham Health Authority Local Research Ethics Committee. The specimens were disaggregated into single-cell suspensions by teasing apart the tissue and fine mincing. Mononuclear cells were isolated by purification over a Lymphoprep gradient (Nycomed Pharma) according to the manufacturer's instructions, aliquoted, cryopreserved, and stored in liquid nitrogen. DNA was isolated from an aliquot of the tonsillar cells for HLA typing by sequence-specific oligonucleotide PCR analysis performed at the Anthony Nolan Trust.

Tonsillar mononuclear cells were infected with KSHV using a protocol similar to one previously described (14). Briefly, Vero cells infected with the recombinant KSHV strain rKSHV.219 (VK219 [15]) were transduced with a pInducer 20 lentivirus (16) engineered to express open reading frame 50 (ORF50) upon addition of doxycycline or ORF50-expressing lentivirus-transduced Vero cells infected with either the BAC16-derived K5 deletion mutant or its paired revertant virus (17) were seeded in 24-well plates at 50,000 cells per well. After 24 h, virus replication was induced for 24 h by the addition of 2 μg/ml doxycycline and 1.25 mM sodium butyrate. The medium was then removed from these wells, and 500,000 tonsillar mononuclear cells were seeded per well, centrifuged onto the VK219 monolayers, and incubated for 48 h. Parallel mock infections of tonsillar cells were conducted either by culturing them on monolayers of VK219 cells that had been treated with 1 mM phosphonoacetic acid (18) for the previous 30 h prior to induction and during the coculture or by culturing the tonsillar cells in the absence of VK219 cells. B cells were then purified from the mock- and KSHV-infected cultures using anti-CD19 Dynabeads (Life Technologies), and the beads were removed using a Detachabead CD19 kit (Life Technologies) according to the manufacturer's instructions.

The purified B cells were cultured in Iscove's minimal essential medium (MEM) with 100 U/ml interleukin 4 (IL-4) (Peprotech), penicillin, streptomycin, gentamicin, and amphotericin B (Fungizone; Life Technologies) on monolayers of L cells transduced to express CD40L that had been gamma irradiated (10,000 rads). After 48 h, the KSHV-infected cells were selected by addition of puromycin to a final concentration between 0.1 and 0.3 μg/ml. The B cells were expanded, moved to fresh L cell monolayers weekly, and maintained under puromycin selection.

The presence of contaminating VK219 cells in the B lymphocyte cultures was determined by performing semiquantitative endpoint PCR assays on 25 ng of DNA extracted from the cultures. The PCR assay detected the neomycin resistance gene carried by the ORF50-expressing lentivirus used to transduce the VK219 cells. The primers used were neo forward (AGG ATC TCC TGT CAT CTC ACC TTG CTC CTG) and neo reverse (AAG AAC TCG TCA AGA AGG CGA TAG AAG GCG). As controls, transduced VK219 cells were mixed with BJAB cells to give 0.1% and 0.01% VK219 cells, and DNA was extracted from these mixtures. These controls and DNA from the samples were subjected to PCR amplification using different numbers of cycles to detect products.

B cell surface marker expression was assessed by staining the cells with antibodies specific to HLA class I, HLA-DR, CD20, CD54, CD86, or appropriate isotype control antibodies (Biolegend). Expression of immunglobulin light chains Igλ and Igκ was assessed by staining cells with biotinylated antibodies specific for these proteins (Southern Biotech) and detected by incubating with avidin-conjugated allophycocyanin (APC)-Cy7 (Biolegend). The cells were fixed in 2% paraformaldehyde (eBioscience) and analyzed on an LSRII flow cytometer (Becton Dickinson), and the data were processed using Flowjo (Treestar).

### Immunofluorescence staining for LANA protein expression in KSHV-infected B cells.

Established KSHV-infected or mock-infected B cell lines were assayed for LANA protein expression by immunofluorescence staining. Cell suspensions were washed in phosphate-buffered saline (PBS), dried on microscope slides, and fixed in cold acetone for 10 min. The slides were then dried, washed in PBS, and stained with either an isotype control rat antibody or a LANA-specific rat antibody (clone LN 53; Advanced Biotechnologies) for 1 h at 37°C. The slides were washed four times, and bound antibody was detected using an anti-rat Alexa 568-conjugated antibody (Life Technologies) with incubation for 1 h at 37°C. The slides were then washed and examined using a Nikon E600 microscope fitted for epifluorescence detection.

### Western blot analysis.

Cells were lysed in 9 M urea with 0.075 M Tris-HCl, pH 7.5, and sonicated, and the lysates were clarified by centrifugation. Protein concentrations were determined by Bradford assay (Bio-Rad Laboratories), and 20 μg of protein was separated by SDS-PAGE and transferred onto nitrocellulose membranes using standard techniques. The blots were probed with antibodies specific for LANA (clone LN53), vIRF3 (CM-A807; Abcam), and actin (AC-74; Sigma-Aldrich). The bound antibodies were detected using the appropriate anti-species peroxidase-labeled antibody, followed by detection using an ECL kit (GE Healthcare).

### Measurement of viral transcripts.

Total RNA was extracted from cells using a NucleoSpin RNA II kit (Macherey-Nagel) according to the manufacturer's instructions. An aliquot (1 μg) was treated with DNase I (Life Technologies) to remove residual genomic DNA before being reverse transcribed using Qscript (VWR).

Selected KSHV transcripts were quantified by TaqMan quantitative reverse transcription (qRT)-PCR using the primer and probe combinations shown in Table 1. The primer and probe sequences were designed using Primer Express 3.0 (Life Technologies) and were based on the BC-1 KSHV genome sequence (accession number U75698). Primer-probe combinations were selected to avoid known KSHV sequence polymorphisms, and assays to detect spliced transcripts were designed to span exon-exon junctions. All TaqMan probes (Eurogentec) were modified with 6-carboxyfluorescein (FAM) and 6-carboxytetramethylrhodamine (TAMRA) at the 5′ and 3′ ends, respectively. Cellular glyceraldehyde-3-phosphate dehydrogenase (GAPDH) mRNA, used as an internal control, was detected using a VIC-labeled commercial assay (Life Technologies; 4310884E).

**TABLE 1:** qRT-PCR assay primer and probe sequences with BC-1 genomic locations

Assay	Sequence (BC-1 genomic location)^*a*^		
	Forward primer	Probe	Reverse primer
LANA/vCyclin/vFLIP	TTTACCTCCACCGGCACTCT (127007–126988)	CACGTCTTCCTCCCCAATCCCTCC (126974–126951)	GTCCCCGGAGACACAGGAT (126927–126945)
vCyclin/vFLIP	GGTAGATGGGTCGTGAGAACACT (123692–123714)	ACCGTCGCCGCTCCGCACTT (123771–123752)	TCCGGCTGACTTATAAACA^AGC (127831–127813^123776–123773)
vFLIP	TTCCACTGCCGC^CTGTAGAG (122848–122859^123595–123602)	TGTCAGGTTCTCCCATCGACGACG (123623–123646)	ACGGACAACGGCTAGCGTACT (123679–123659)
vIL-6	GATGCTATGGGTGATCGATGAA (17753–17732)	TTCCGCGACCTCTGTTACCGTACCG (17728–17704)	GGGCTCTAGAATACCCTTGCAGAT (17678–17701)
vIRF3	GGAGAAGACCA^GGCCATTTG (90952–90942^90847–90838)	TGAGGAGGATCACCCAGCCTTTTGC (90815–90791)	CTGCGTGACCGGCACAT (90773–90789)
ORF50	GCAAGATGACAAG^GGTAAGAAGC (71601–73613^72572–72581)	CTGTGTGGAAAGCTTCGTCGGCCTC (72592–72616)	TGGTAGAGTTGGGCCTTCAGTT (72645–72624)
K3	GCGGGTTGAAGTGTTTCCAT (19139–19120)	TCGGCCGACATCACCAGAGTGTG (19112–19090)	TTTCCTGAAGCTCGATCTCCTCTA (19063–19086)
K5	TCCACCCGCAGTGTTTAAGC (26368–26349)	TGTCTCGAAACACGGCCTGTCAAATG (26335–26310)	CGTGCGCGTGCGGTATA (26283–26299)

a ^, splice junction.

Amplification reactions were prepared in a final volume of 25 μl containing 1× TaqMan Universal MasterMix II (Life Technologies), 300 nM forward and reverse primers, 200 nM probe, 0.5 μl GAPDH reagent, and 5 μl cDNA. PCR amplifications were performed using an ABI 7500 with default thermocycling conditions. All test samples were run in duplicate, while template-negative and RT-negative samples served as controls. To determine the absolute levels of KSHV and GAPDH transcripts, serial dilutions from 1 to 10^5^ copies of a plasmid (AQ2) were included in each PCR experiment and used to generate appropriate standard curves. AQ2 was derived from the AQ plasmid (19) by the insertion of a commercially synthesized 1,093-bp sequence carrying the contiguous KSHV amplicons (GenScript). All data were analyzed using Sequence Detection Software v2.0 (Applied Biosystems) and are reported as copies relative to GAPDH.

Comparisons of gene expression levels between PELs and infected B lymphocytes were performed using the R statistical program (v 3.0.2) (20) on log_10_-transformed mean values so that they were normally distributed as judged using the Shapiro-Wilk test. These transformed values were then subjected to two-sample *t* tests to determine differences in transcript levels between PEL lines and infected lymphocytes.

### KSHV genome loads.

DNA was extracted from cells using a NucleoSpin Tissue kit (Macherey-Nagel), and viral-genome loads were determined by quantitative PCR (qPCR). KSHV DNA was detected using the viral IL-6 (vIL-6) primer-probe combination, while cellular beta 2 microglobulin (B2m), used as an internal control, was detected using primers described previously (21). Serial dilutions of AQ2 plasmid and BJAB cell DNA were used to generate standard curves for vIL-6 and B2m, respectively. Data are expressed as KSHV genome copies per cell, assuming two B2m genes per diploid cell.

### T cells and recognition experiments.

The ability of T cells to recognize KSHV-infected targets was determined as described previously, using established T cell clones (6). Briefly, triplicate cultures of 5,000 T cells were incubated with 50,000 target cells that were either KSHV-infected or mock-infected target B cells or B cells sensitized with the T cell cognate synthetic-peptide epitope (Mimotopes). The cells were incubated in RPMI 1640-10% fetal calf serum (FCS) for 18 h, and the supernatants were harvested from these cultures and assayed for gamma interferon (IFN-γ) by enzyme-linked immunosorbent assay (ELISA) (Endogen).

## RESULTS

### KSHV infection of primary B cells and their propagation.

**FIG 1:**
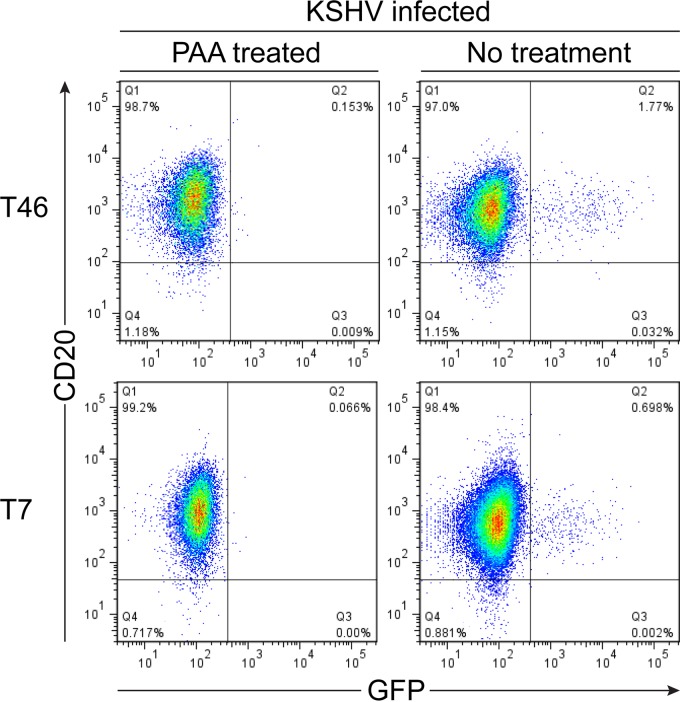
Frequency of KSHV-infected B cells after *ex vivo* infection. Tonsillar B cells from donors T46 and T7 were either infected with KSHV by culturing on monolayers of induced VK219 cells for 48 h or mock infected by culturing on induced monolayers that had been pretreated with phosphonoacetic acid (PAA) to inhibit virus replication. The B lymphocytes were then magnetically sorted, cultured for a further 72 h to allow expression of the GFP reporter from the rKSHV.219 genome, and then stained for CD20 surface expression, and the proportion of cells expressing GFP as a marker of infection was determined by flow cytometry.

In a preliminary set of experiments, we determined whether we could infect tonsil-derived B cells with rKSHV.219 virus. Unfractionated tonsillar mononuclear cells were infected with KSHV by incubating them on monolayers of Vero cells that contained latent rKSHV.219 that had been treated 24 h previously to induce virus replication. As a mock infection, parallel aliquots of tonsillar cells were incubated on monolayers of induced VK219 cells that had been treated for the previous 30 h with phosphonoacetic acid to inhibit virus production. After 48 h of coculture, CD19-expressing B cells were selected and cultured for 72 h to allow green fluorescent protein (GFP) expression from the rKSHV.219 genome, and the proportion of infected cells was identified by flow cytometry. Figure 1 shows two representative results of such infections from tonsillectomy patients T46 and T7. Consistent with previous reports (9), we found that these cells could be infected at a low percentage; typically, GFP-expressing cells would be detected in the range of 0.5% to 1.6% of B cells.

**FIG 2:**
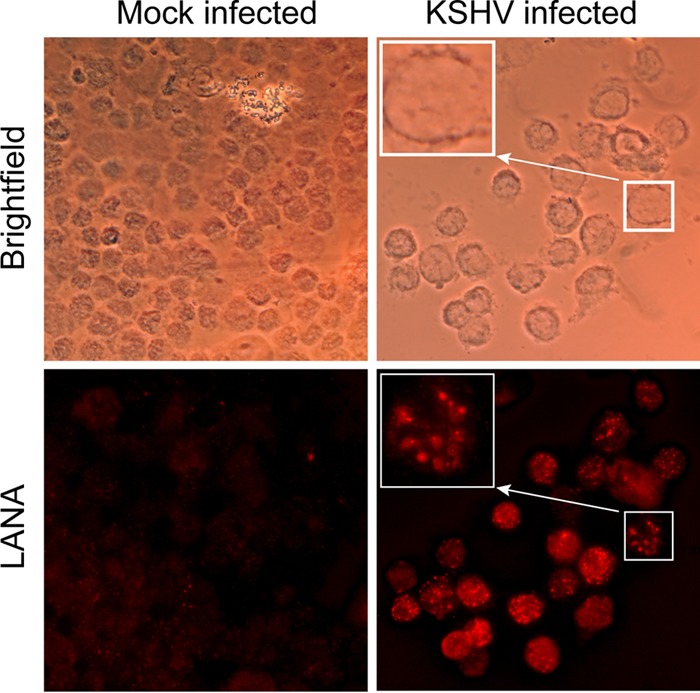
Expression of LANA protein in B cell lines by immunofluorescent-staining analysis. Mock-infected or KSHV-infected cells that had been established for 8 weeks were stained with a LANA-specific monoclonal antibody, and bound antibody was detected by staining with an Alexa-fluor conjugated secondary antibody. Antibody binding was visualized by epifluorescence microscopy. Boxed region indicates a magnified image of a LANA-staining cell.

We next asked whether the infected cells could be expanded by *in vitro* culture. Previous studies had shown that, unlike the related gammaherpesvirus EBV, *ex vivo* infection of B cells with KSHV does not lead to transformation of the B cells (9) but induces some limited proliferation (10). To expand the population of B cells, we delivered a mitogenic stimulus to them by incubating them on CD40 ligand-expressing L cells in the presence of IL-4. As rKSHV.219 virus carries a puromycin resistance gene, we enriched for infected B cells by selection with puromycin. We found that the infected cells were very sensitive to puromycin, requiring low doses to enrich the cells, which did not obviously affect L cell viability. However, compared to mock-infected B cells cultured in parallel without puromycin, the infected cells grew slowly, doubling every 4 to 5 days compared to the parallel uninfected B cells, which doubled every 72 h. All subsequent experiments used cells that had undergone at least 3 to 4 weeks of selection and that contained between 5% and 50% GFP-expressing cells. The presence of contaminating VK219 cells was examined using a semiquantitative PCR assay to detect the neomycin resistance gene carried by the ORF50-expressing lentivirus used to transduce these cells. Only DNA extracted from cells derived from two of nine lines, T44b and T48, had detectable sequence at levels less than 0.01% of the population (data not shown). B cell lines could be maintained for a similar length of time as mock-infected cells, typically for up to 12 weeks, with some lasting >20 weeks. During passaging of these cells, we monitored for red fluorescent protein (RFP) expression, the gene for which in rKSHV.219 is expressed under the control of the PAN promoter, as a marker of lytic-cycle replication and observed few cells, usually less than 5%, expressing this marker in the cultures. To confirm that the B cells were infected with rKSHV.219, we purified them by fluorescence-activated cell sorting (FACS) for GFP expression and conducted immunofluorescence assays on the cells, staining for LANA. Figure 2 shows representative results from one of three sorts, showing the characteristic punctate staining of LANA, reminiscent of the pattern seen in primary effusion lymphomas (22), indicating these KSHV-infected cells could be expanded *in vitro*.

### Antigen and gene expression of KSHV-infected primary B cells.

**FIG 3:**
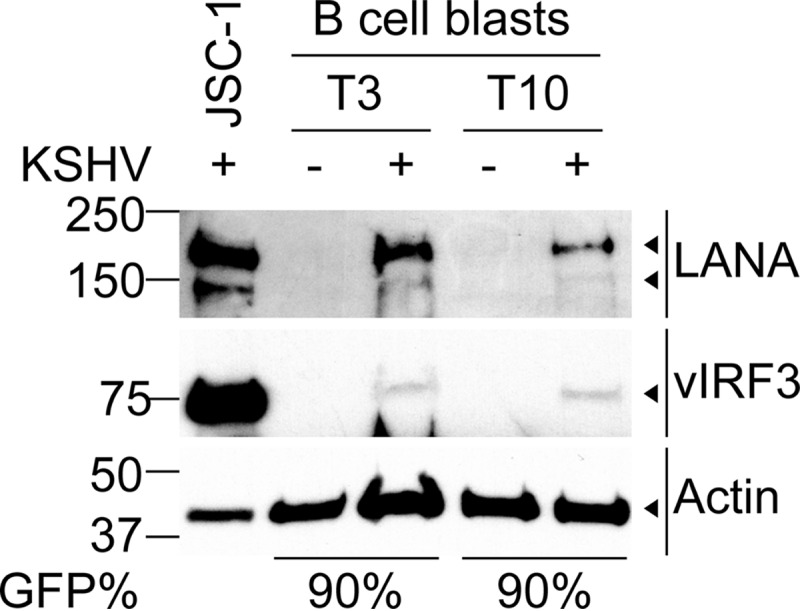
Western blot analysis for detection of viral proteins in lysates from B cell lines. Proteins from lysates of the PEL JSC-1 line and mock-infected and KSHV-infected B cell lines that had been maintained for 18 weeks were separated by PAGE and blotted onto polyvinylidene difluoride (PVDF) membranes. The blots were sequentially probed for either LANA, vIRF3, or actin. The arrowheads indicate specific protein detection.

In the next series of experiments, we sought to determine which KSHV genes were expressed in the infected B cells, principally to identify which could be used as immunological targets and also to monitor any immune evasion gene expression. We focused on likely latent genes and their products, as we observed few RFP-expressing cells in our established cultures, suggesting no substantial spontaneous lytic reactivation was occurring. Western blot analysis was performed on lysates from two tonsillar preparations of rKSHV.219-infected B cells that had been sorted by FACS to 90% purity or mock-infected B cells maintained on CD40 ligand and IL-4 in parallel. As a control, a lysate from the PEL line JSC-1, from which rKSHV.219 was derived, was used. Blots were initially probed with antibodies to vCyclin and vFLIP; however, no expression of these proteins was detected in the JSC-1 or any B cell lysates, nor could we detect expression of the EBV protein EBNA1, found in approximately 80% of PEL lines, in infected B lymphocyte lysates (data not shown). The blots were then sequentially probed for LANA, vIRF3, and actin, the last as a loading control, and the results are shown in Fig. 3. Infected B cells demonstrated expression of LANA, although at levels lower than those seen in JSC-1, while vIRF3 expression in the infected B cells was substantially lower than that in the PELs.

**FIG 4:**
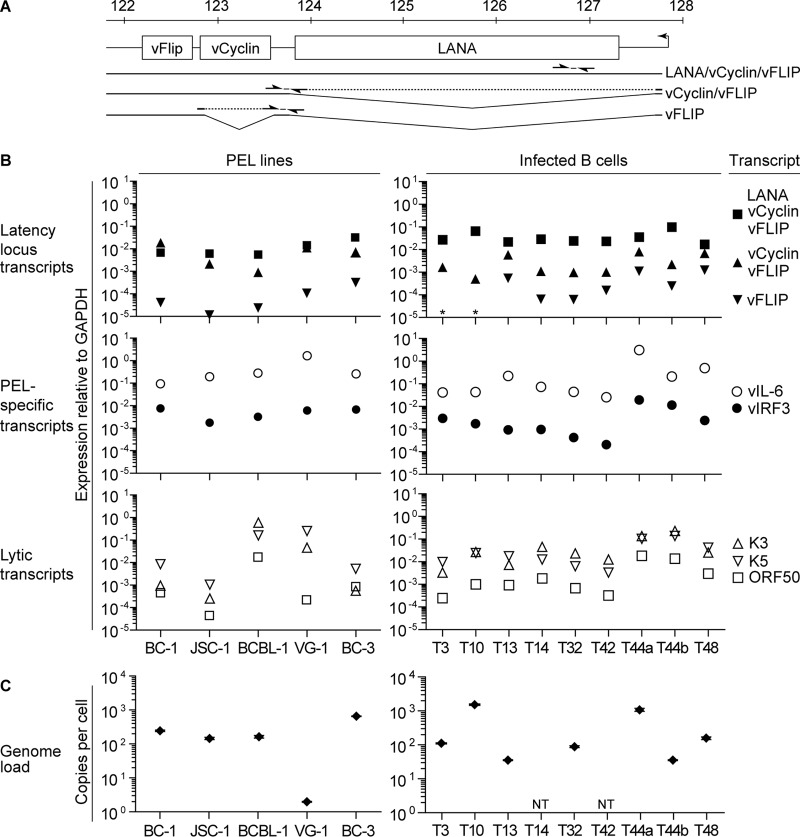
Gene expression analysis and genome loads within KSHV-infected cell lines. (A) Map of qPCR assays to detect splice variants driven from the constitutively active promoter of the latency locus. The arrows represent primers, and the dotted lines indicate intronic regions spanned by the primers. (B) Gene expression was determined from cDNA prepared from the PEL lines BC-1, JSC-1, BCBL-1, VG-1, and BC-3 and the infected B cell lines and assayed for expression of the following transcripts: tricistronic LANA-vCyclin-vFLIP, bicistronic vCyclin-vFLIP, monocistronic vFLIP, vIL-6, vIRF3, K3, K5, ORF50, and GAPDH. The transcripts are expressed relative to GAPDH transcript abundance. The asterisks indicate <10^−5^ vFLIP transcript detected for T3 and T10. (C) The genome load was determined in DNA extracted from the cell lines in panel B and quantified using the vIL-6 qPCR assay. The results shown for the infected B cell lines have been corrected to represent 100% GFP expression. Infected lymphocytes had been established for the following times: T3, 18 weeks; T10, 18 weeks; T13, 10 weeks; T14, 8 weeks; T32, 10 weeks; T42, 12 weeks; T44a, 10 weeks; T44b, 8 weeks; and T48, 6 weeks.

To further characterize these infected B cells, we measured viral transcript expression by qRT-PCR using a panel of TaqMan-based assays we developed. They included the splice variants of transcripts from the major latency locus driven from the constitutive promoter, as shown in Fig. 4A (23, 24), namely, the tricistronic mRNA encoding LANA, vCyclin, and vFLIP; the bicistronic mRNA encoding vCyclin and vFLIP; and the monocistronic mRNA encoding vFLIP. The specificity of assays that detect spliced transcripts was confirmed by testing against unspliced (genomic) DNA, which showed no detection using the bicistronic vCyclin-vFLIP assay and weak detection using the monocistronic vFLIP assay, giving a background of 0.13% compared to the unspliced tricistronic message. Assays measuring the PEL-expressed vIRF3 and vIL-6 mRNAs were also developed, as were assays for three lytic-cycle-expressed genes, including the immediate early expressed lytic switch gene ORF50 and two early expressed mRNAs, K3 and K5. The locations of the amplicons within the genome, primer, and probe sequences are shown in Table 1. To validate these assays, we tested them on RNA extracted from the PEL lines BC-1, JSC-1, BCBL-1, VG-1, and BC-3. All the assays were compared to a standard, which was a synthetic plasmid containing the PCR amplicons, thereby allowing absolute quantitation of transcript levels within a cell type.

Figure 4B (left) shows the results of qRT-PCR assays reporting results relative to GAPDH expression, grouping transcripts into those expressed from the latency locus (LANA, vCyclin, and vFLIP), PEL-specific transcripts (vIRF3 and vIL-6), and lytic transcripts (ORF50, K3, and K5). Latency locus transcripts showed in most cases that the tricistronic transcript was the most abundant, followed by the bicistronic transcript, the exception being BC-1, where this pattern was reversed. Previous Northern blot analysis of BC-1 showed a profile consistent with what we detected, but with some contrast for BCBL-1, where Northern analysis indicated the bicistronic transcript was more abundant than the tricistronic message (23, 25). What dictates splicing of these RNAs is unknown, so why this difference occurred is not clear, but it may be related to drift in gene expression patterns in cultured cells over time or to the culture conditions used in our experiments. Interestingly, the BCP-1 PEL, not analyzed in our experiments, showed in Northern analysis a pattern of transcript expression similar to what we detected in most PELs, with the tricistronic message more abundant than the bicistronic message (26). Low-level expression of the monocistronic transcript was detected, consistent with previous findings (24). All the PELs expressed vIL-6 RNA, usually at high levels compared to other transcripts, and all expressed vIRF3. In most cases, low levels of the ORF50 transcript were detected, but perhaps surprisingly, transcripts for the early expressed K3 and K5 genes were detected.

We next performed these qRT-PCR assays on RNA extracted from the infected B cells that had been enriched for infection by selection with puromycin. These cells showed different levels of KSHV infection, as judged by GFP expression: T3, 90%; T10, 90%; T13, 75%; T14, 43%; T32, 51%; T42, 62%; T44a, 51%; T44b, 54%; and T48, 29%. To account for these variations and to allow comparison of transcript levels between the different infected cells and PELs, we corrected the transcript levels to 100% GFP expression. Figure 4B (right) shows the transcript levels in these cells, where patterns of transcript expression showed similarities to what was seen in the PELs. Thus, the tricistronic was the most abundant of the latency locus transcripts, followed by the bicistronic, with very low levels of the monocistronic transcripts detected. The vIL-6 transcripts were again the most abundant detected, while there was lower and variable expression of vIRF3. Low levels of ORF50 transcript were detected, consistent with our observation of few if any RFP-expressing cells in the cultures. However, similar to the PEL data, some expression of K3 and K5 transcripts was detected in the infected B cells. Statistical analysis comparing individual transcript levels between PELs and infected lymphocytes showed that only the tricistronic transcript showed a significant difference between the two cell types (*t* test; *P* = 0.023). Overall, the pattern of transcript levels in the infected B cells broadly recapitulated what was detected in the PELs.

PEL lines are known to harbor high frequencies of KSHV genomes, so using our qPCR assays, we quantified the genome load per cell in the infected B cells and compared these levels to what we observed in PELs. Figure 4C shows the results of these assays. PELs contained variable but high levels of genomes, with BC-3 containing over 600 copies of the genome while JSC-1, BCBL-1, and BC-1 had lower yet substantial frequencies of genomes, between 145 and 244 copies per cell. These values were comparable to those previously reported using a similar methodology for these cell lines (27). Interestingly in repeated assays on VG-1 DNA, only two copies of the genome per cell were detected. Analysis of the genome loads of infected B cells, corrected for the frequency of GFP-expressing cells, showed they had lower frequencies than most PELs in five of seven lines analyzed, while lines derived from donors T10 and T44a showed comparable or greater numbers of copies of genomes. A second, independently established line from the second donor, T44b, showed levels closer to those of the other infected cells. These findings suggest that the infected B cells maintain similar or reduced genome loads compared to PELs.

### Cell surface phenotype analysis of infected B cells.

**FIG 5:**
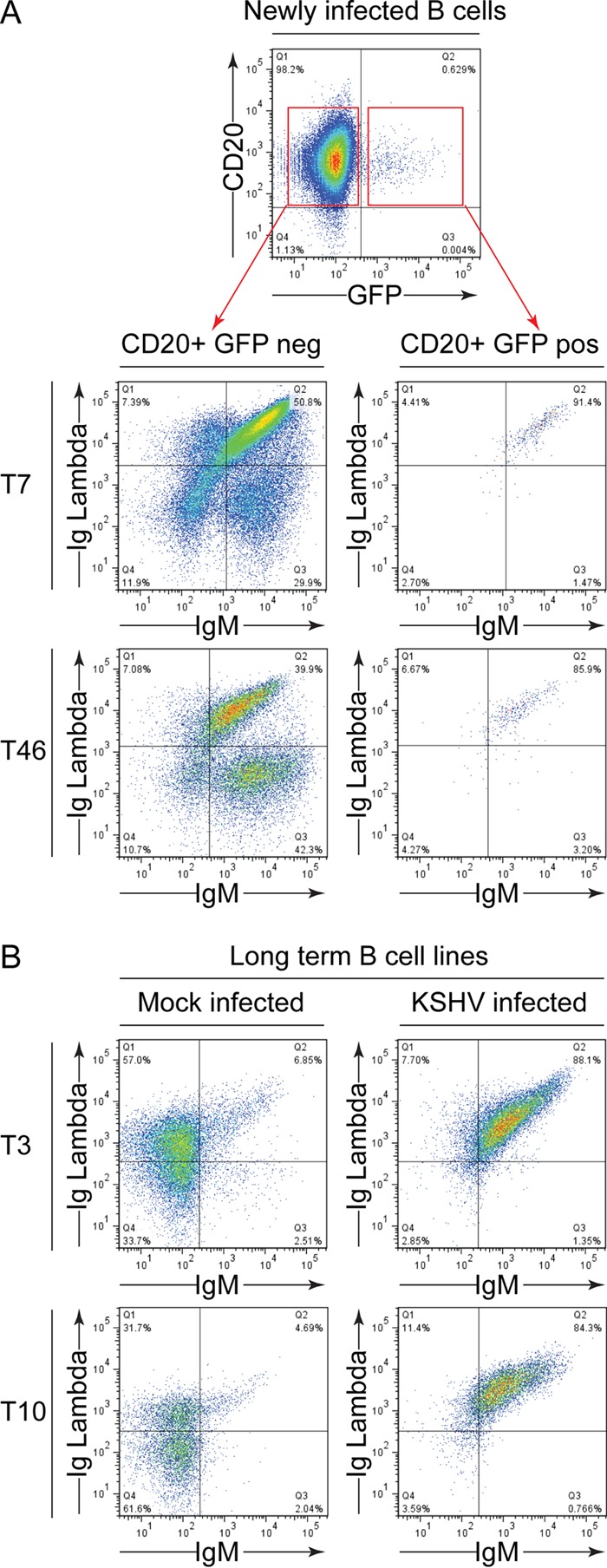
Cell surface immunoglobulin usage by KSHV-infected B lymphocytes. (A) Tonsillar cells were infected with KSHV as described for Fig. 1 and stained for expression of CD20, Igλ, and IgM, followed by flow cytometry analysis. The populations shown are gated on the CD20 GFP-negative uninfected cells or the CD20 GFP-positive infected population. (B) B cell lines that were either mock or KSHV infected and maintained for 20 weeks were analyzed for cell surface expression of Igλ and IgM. The mock-infected cells were gated on viable cells, while the KSHV-infected cells were gated on GFP viable cells.

We next examined the B cells to identify phenotypic markers associated with infection and whether there was evidence of altered immunological marker expression. First, the identity of surface immunoglobulin heavy and light chain expression was determined, as in MCD, KSHV is found in IgM-positive λ light-chain-expressing cells (28). Here, purified B cells that had been exposed to the virus producer cells and then cultured for 72 h to allow GFP expression were stained for surface expression of immunoglobulin light chain type and IgM. Figure 5A shows flow cytometry results of this analysis, where GFP-negative uninfected cells showed there was split expression of Igλ and Igκ (not shown) and that the majority of the population expressed IgM. In contrast, few GFP-positive cells showed Igκ expression (data not shown); however, there was a marked if not exclusive preference for coexpression of Igλ and IgM by the infected cells. Figure 5B shows a similar analysis of established B cell lines that had been maintained for more than 5 months. Mock-infected cell lines contained both Igλ- and Igκ (not shown)-expressing cells, but there were few IgM-expressing cells in these cultures. The KSHV-infected cells, however, showed little evidence of Igκ expression (not shown) but expressed Igλ and had maintained their expression of IgM, indicating that culturing these cells in this fashion recapitulates phenotypic features seen in KSHV disease states.

**FIG 6:**
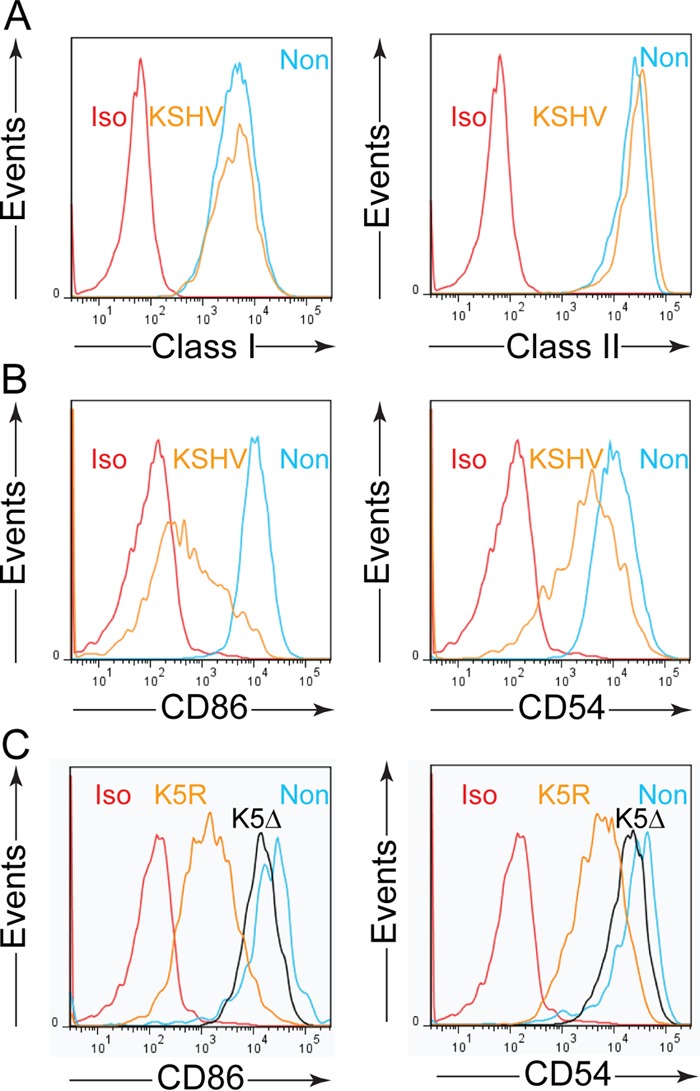
Flow cytometric analysis of cell surface-expressed immunological markers on KSHV-infected B cell lines. (A) KSHV-infected B cell lines that had been established for 8 weeks were stained with antibodies specific for either MHC class I or class II or an isotype control antibody (Iso) and gated on either GFP-positive infected cells (KSHV) or GFP-negative cells coresident in the culture (Non). (B) KSHV-infected B cell lines were stained for expression of CD86 or CD54 and gated on infected and coresident noninfected cells as for panel A. (C) KSHV-infected cell lines that had been established for 6 weeks were infected with either a virus with K5 deleted (K5Δ) or a virus derived from this construct in which the K5 gene was restored (K5R) and stained for expression of CD86 or CD54 on infected and coresident noninfected cells as for panel A. The data are representative of at least three independent cell lines for each assay.

KSHV carries genes such as those encoding K3, K5, and vIRF3, which can modulate cell surface expression of molecules that immune effectors recognize or receive costimulatory signals from (7, 29–32). As such, we determined the cell surface expression of HLA class I, class II, and the costimulatory molecules CD86 and CD54 (ICAM) on infected cells that had undergone enrichment by puromycin selection and compared their expression to that in coresident uninfected cells within the culture by flow cytometric analysis. Despite expression of K3 transcripts in infected cells (Fig. 4), no obvious modulation of the protein's target, namely, surface HLA class I, was observed (Fig. 6A). Similarly, infected cells showed little change in surface levels of HLA class II compared to uninfected cells, despite the presence of vIRF3 transcripts.

Analysis of T lymphocyte costimulatory marker expression is presented in Fig. 6B, where, compared to uninfected cells, infected cells had reduced surface levels of CD86 and CD54. Such a profile is consistent with expression of the K5 gene as measured in the qRT-PCR analysis, whose product has ubiquitin ligase activity that induces endocytosis of these proteins. To test whether K5 was responsible for the downregulation of these markers, B cells were infected with a recombinant KSHV in which the K5 gene was deleted (K5Δ) or a derivative of this virus in which the K5 gene was restored (K5R) (17). Cells were selected, and surface marker analysis was conducted as before; Fig. 6C shows CD86 and CD54 surface expression analysis results for one of three pairs of cell lines infected with the K5Δ or K5R virus. Cells infected with the K5Δ virus showed substantial but not complete restoration of surface CD86 and CD54 expression compared to coresident uninfected cells, suggesting that K5 expression alters at least some surface markers on infected cells in this model.

### Recognition of KSHV-infected B cells with KSHV-specific CD4^+^ T cells.

**FIG 7:**
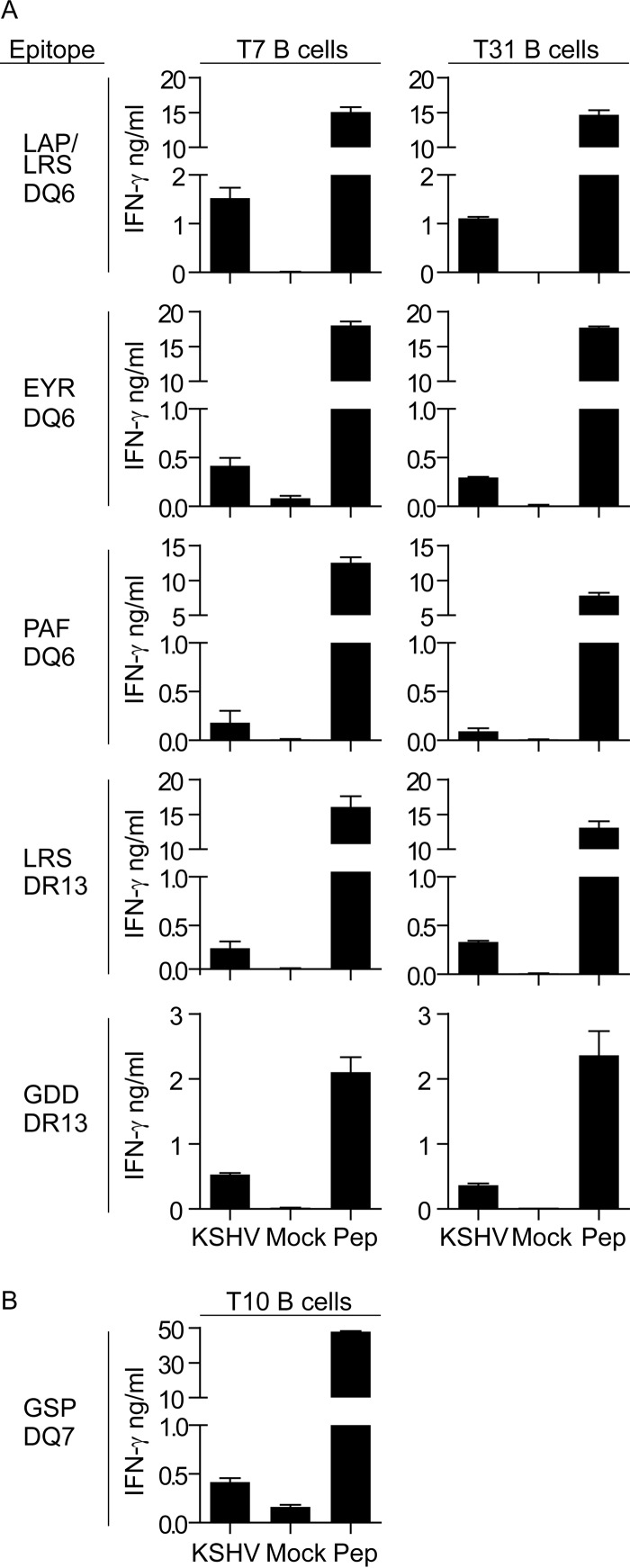
LANA-specific CD4^+^ T cell recognition of mock- or KSHV-infected B lymphocyte lines. (A) KSHV-infected B cell lines, mock-infected B cell lines, or mock-infected B cell lines sensitized with the T cell cognate peptide (Pep) derived from donors T7 and T31 that coexpressed HLA-DQ6 and HLA-DR13 were challenged with the LANA-specific HLA-DQ6-restricted clones LAP/LRS, EYR, and PAF or the DR13-restricted clones LRS and GDD. The B lymphocyte lines had been established for 10 weeks. (B) KSHV-infected or mock-infected B cell lines derived from donor T10 that expressed HLA-DQ7 were challenged with the LANA-specific HLA-DQ7-restricted clone GSP. The B lymphocyte lines had been established for 8 weeks. In both cases, T cell recognition of targets was assessed by measuring IFN-γ secretion. The error bars represent the standard deviations of assay replicates.

As we had demonstrated that the KSHV-infected cells expressed LANA RNA and protein but did not appear to obviously modulate MHC class II cell surface expression, we determined whether these infected cells could be targeted by a panel of LANA-specific CD4^+^ T cells. We were especially interested in the ability of CD4^+^ T cells to recognize this antigen, as it is expressed in all infected cells *in vivo* and the repeat sequences within LANA have been shown to decrease presentation of epitopes to CD8^+^ T cells (33, 34). For these experiments, we HLA typed the infected B cells and challenged them with HLA-matched CD4^+^ T cell clones specific for LANA that we had previously established (6). A total of six T cell clones were used, three of which were responsive to the HLA-DQ6-presented peptide LAPSTLRSLRKRRLSSPQGP (LAP/LRS), EYRYVLRTSPPHRPG (EYR), or PAFVSSPTLPVAPIP (PAF); two of which were specific for the HLA-DR13-presented peptide LRSLRKRRLSSPQGP (LRS) or GDDLHLQPRRKHVAD (GDD); and one of which was responsive to the HLA-DQ7-presented peptide GSPTVFTSGLPAFVS (GSP). Here, the clones were incubated with either the infected B cells, mock-infected B cells, or mock-infected B cells sensitized with the T cell cognate peptide for 18 h, and recognition was assessed by measuring IFN-γ secretion from the T cells. Figure 7 shows representative results from three assays in which the effectors were challenged with B cell lines infected with KSHV or mock-infected cell lines that had been maintained on CD40L and IL-4 in parallel. T7 and T31 KSHV-infected targets were 68% and 93% GFP positive, respectively, and they coexpressed both HLA-DR13 and HLA-DQ6, while T10-infected targets were 23% GFP positive and expressed HLA-DQ7. In all cases, we observed IFN-γ secretion from the T cells when challenged with the infected cells, indicating that despite the downregulation of cell surface costimulatory molecules and the low-level expression of vIRF3, these KSHV LANA-specific CD4^+^ T cells could recognize the KSHV-infected B cells.

## DISCUSSION

In the present study, we developed a model of primary B lymphocyte infection with KSHV to examine gene expression and immune recognition of infected B cells, which unlike PEL lines, have not gone through a transformation process. Infected B lymphocytes expanded with mitogenic stimuli expressed latent proteins and genes in a pattern similar to what is observed in PELs. Phenotypically, these recapitulated features of MCD-infected cells, namely, the almost exclusive expression of IgM and Igλ by the infected cells. Although these cells expressed genes associated with immune evasion, some of which did modulate cell surface markers, we found that the KSHV-infected cells were capable of being recognized by LANA-specific CD4^+^ T cells.

Our initial studies characterizing the KSHV-infected B cells showed that they expressed key proteins, namely, LANA and vIRF3, while qRT-PCR analysis indicated that a repertoire of genes similar to those seen in PELs assayed in parallel were expressed. However, despite finding transcripts for LANA and vIRF3 in the infected lymphocytes, the corresponding levels of protein for each were lower than in the PELs, suggesting that in this model, factors other than transcript abundance affect protein levels. We have previously found that ectopic expression of LANA or vIRF3 in model cell lines at levels similar to those in PELs is toxic (unpublished observations). We speculate that PELs adapt to high-level expression of these proteins during the transformation process and that the infected lymphocytes in the present study had not had sufficient time to adapt to high-level protein expression. Some expression of the lytic-cycle-assigned K3 and K5 genes was also observed, potentially related to the low-level expression of ORF50. However, few cells expressed RFP, a marker of lytic cycle replication, suggesting that either these transcripts were detected from the few cells with lytic virus replication or the genes were being expressed outside true lytic replication. The latter case seems most likely, as all the infected lymphocytes, rather than a subset, showed decreased surface expression of K5 target proteins. Furthermore, recent transcriptome analysis of the PEL BCP-1 indicated that some transcripts, particularly K5, can be found in these cells in the absence of obvious lytic cycle replication (35). Such expression patterns show some contrast to what has been observed in latently infected SLK cells, where there is minimal expression of K5 and modulation of its targets outside lytic cycle replication (17). K5 expression, then, may have been induced in our cells due to the culture conditions used, or, as PELs express this transcript, the K5 promoter may be activated in a B cell background due to B cell-specific transcription factors. Alternatively, this may be a consequence of the elevated genome loads detected, as high genome loads, at least in primary infection of cells, are associated with the modulation of these cell surface immune-cell-stimulating ligands (36).

Despite the expression of K5 and the decreased surface expression of CD86 and CD54, no obvious modulation of surface MHC class I was seen on the infected cells. This is likely due to the apparent increased sensitivity of CD86 and CD54 to K5 compared to MHC class I, especially certain allotypes of class I (29, 31, 37). Turning to MHC class II expression, no obvious decrease in cell surface expression of these proteins was observed on infected cells despite the expression of vIRF3. This protein can block promoter activity of the class II transcriptional transactivator CIITA, which is needed for expression of MHC class II and other genes required for the MHC class II-processing pathway (7). Although vIRF3 transcript was expressed, we detected low levels of protein in infected cells compared to the JSC-1 PEL. Interestingly in this context, we have previously found that BCBL-1 PELs similarly express low levels of vIRF3 protein compared to other PELs but maintain good expression of surface MHC class II (6).

Analysis of cell surface marker expression showed that the vast majority of KSHV-infected cells were predominantly within the Igλ light chain/IgM heavy chain subset, reminiscent of the features observed within infected cells of MCD lesions (28). Established cultures of infected cells maintained this phenotype, while parallel mock-infected cultures switched immunoglobulin heavy chain isotype usage, a likely consequence of continuous CD40 ligand and IL-4 stimulation, suggesting that virus infection suppresses isotype switching. Interestingly, this bias of light chain usage was seen within 72 h after virus infection. Related studies in which KSHV infection of tonsillar B lymphocytes was examined at 72 h postexposure for LANA protein expression have also shown that the vast majority of LANA-expressing cells were in the Igλ-using population (10). Why this bias occurs is unclear, as there are no obvious functional or phenotypic differences between the two subsets. Potentially, this selection may be a consequence of vFLIP expression, as B cells in a transgenic vFLIP mouse model more frequently utilize the light chain (38). In this case, the ability of vFLIP to promote expression of NF-κB may be responsible, as this transcription factor is required at key stages of immature B cell development for the selection of Igλ-using cells (reviewed in reference 39). However, as light chain rearrangement occurs during B lymphocyte maturation in the bone marrow, the tonsillar B lymphocytes used in this study likely had already undergone light chain selection. This may suggest that infected cells may be converted to Igλ usage through processes such as receptor revision (40), although this may seem unlikely given that the vast majority of cells express this receptor by 72 h postinfection. Alternatively, Igκ-using cells may not sustain infection.

The T lymphocyte response is vital for effective control of KSHV infection and disease; however, whether one subset of T lymphocytes, either CD4^+^ or CD8^+^, is more effective remains an open question. This is particularly relevant when examining features of proteins such as the genome maintenance protein LANA, which is expressed in all KSHV-infected cells and malignancies, making it an attractive immunological target. Like its related genome maintenance protein homologue in EBV, EBNA1, LANA includes extensive repeat sequences, some of which share nucleotide homology with EBNA1. They function to limit protein synthesis in *cis* and to inhibit proteasomal degradation (34, 41–43). As most CD8^+^ T cell epitopes appear to be derived from newly synthesized proteins that are degraded by the proteasome (44), these features of LANA have been shown to minimize CD8^+^ T cell recognition of model epitopes inserted into LANA (33, 34, 41). Although LANA-specific CD8^+^ T cell recognition of KSHV-infected cells is untested, one may predict that recognition is minimal. In contrast, epitope generation for CD4^+^ T cell recognition would not be subject to these restrictions, and as B lymphocytes express class II MHC, they may be legitimate targets for LANA-specific CD4^+^ T cells. Indeed, we found that for all T cell specificities tested, infected cells could be recognized by the MHC-matched T cell clones. The magnitudes of responses are comparable to those seen in analogous experiments using EBV-specific CD4^+^ T cells targeting epitopes from some latent antigens, such as the EBNA3 proteins expressed by EBV-transformed B lymphoblastoid cell lines (LCLs) (45–48).

The ability of the LANA-specific CD4^+^ T cell clones to recognize KSHV-infected cells shows some contrast to what is seen using EBV EBNA1-specific CD4^+^ T cells challenged with their cognate LCL, which naturally expresses EBNA1. These effectors either do not recognize or weakly recognize such targets (47, 49). This is thought to be a consequence of the poor release of EBNA1 protein by LCLs, thereby restricting the ability of cells to take up antigen and re-present it to T cells or making EBNA1 epitope presentation reliant on endogenous processing of antigen through a macroautophagy route (48, 50, 51). We have previously shown that B cells with intact antigen-processing pathways exogenously fed LANA protein can take up, process, and present epitopes to LANA-specific CD4^+^ T cells and that such B cells can also present endogenously expressed LANA (6). These findings suggest that LANA, despite performing a similar function and having some sequence homology to EBNA1, is processed and presented differently and may be a more relevant target for CD4 T cells than EBNA1 in EBV infection. Furthermore, we suggest that as LANA has properties that limit efficient CD8^+^ T cell recognition, CD4^+^ T cell immunity to the protein is likely to be important in control of KSHV infection and disease.
